# Editorial: Application of PROTACs as a Novel Strategy for Drug Discovery

**DOI:** 10.3389/fchem.2021.740196

**Published:** 2021-07-30

**Authors:** Hany S. Ibrahim, Wolfgang Sippl

**Affiliations:** ^1^ Department of Medicinal Chemistry, Institute of Pharmacy, Martin-Luther-University of Halle-Wittenberg, Halle, Germany; ^2^ Department of Pharmaceutical Chemistry, Faculty of Pharmacy, Egyptian Russian University, Cairo, Egypt

**Keywords:** PROTACs, E3 ligase inhibitors, protein degradation, heterobifunctional molecules, ubiquitin-proteasome system

Targeted protein degradation in cells by novel chemical compounds is seen as one of the emerging techniques to establish alternatives to classical small molecule-based protein inhibition. Chemically induced protein degradation can serve as a biological tool and also offers new opportunities for drug discovery. One series of compounds that has been established for protein degradation are the so-called PROTACs. These compounds are heterobifunctional molecules consisting of three parts: an E3 ligase ligand, a binder for the target protein of interest (POI) and a linker that connects them. The E3 ligase ligand of PROTACs hijacks the ubiquitin-proteasome system, and brings the E3 ligase into spatial proximity to the target protein to degrade it. Although this is a brilliant idea to design new active molecules, there are still many obstacles in the development of novel PROTACs. This special issue features several manuscripts discussing recent advances in PROTAC design and showing how effective PROTACs can be developed. In addition, the challenges faced by PROTACs compared to small molecule inhibitors are discussed.

In the contribution of Xu et al. the PROTAC strategy was exploited to knock out the liver X receptor LXRβ which belongs to the class of nuclear hormone receptor. LXR antagonists are considered as promising treatments for hypercholesterolemia and diabetes. Using the reported agonist GW3965, PROTACs based on the E3 ligase ligands pomalidomide and VH032 were synthesized and both series were compared in terms of target receptor degradation activity. PEG chains with different lengths were used to connect the agonist GW3965 and the E3 ligase ligands. The degradation was confirmed by Western blot studies, and the cellular effect of LXRβ PROTACs on Huh7 cells was studied. It was found that the combination of the VHL ligand VH032, a PEG-5 linker and GW3965 as a binder for the target protein showed the best degrading activity among the synthesized PROTACs.

The review by Cecchini et al. gives a detailed overview about the basic concepts related to protein degradation by PROTACs. The authors cover various aspects including design, pharmacology and thermodynamic challenges connected with PROTAC development. A particular focus is on available strategies to enhance cellular uptake, including suggestions of promising biological tools for the *in vitro* evaluation of PROTACs permeability. This review article is highly recommended as an introduction to the challenges and solutions of PROTAC design and development.

In the review published by Bricelj et al. a broad overview on synthetic concepts that have been used in various PROTAC development projects is given. In addition to the E3 ligases CRBN and VHL, the review also covers the less commonly studied IAPs and MDM2. The authors describe and compare different preparative routes to E3 ligase ligands with respect to feasibility and productivity. A particular focus is set on the chemistry of the linker attachment by discussing the synthetic opportunities to connect the E3 ligase ligand at an appropriate exit vector with a linker to assemble the final PROTAC. For each PROTAC class the authors give a statistical analysis of how often the various building blocks are used for the synthesis.

Zeng et al. publish a review article discussing the different types of photoPROTACs, either photo-switchable PROTACs or photo-encapsulated PROTACs, and the scientific basis for designing such types. PhotoPROTACs enables the reversible on/off switching of protein degradation. In a separate section, the principles of photoPROTACs and their biological significance were discussed. This review showed an interesting comparison between the biological activity of some known PROTACs such as ARV-771 and their photoPROTAC analogues. Furthermore, the review highlights the further challenges in the development of photoPROTACs.

The review by Yu et al. focuses on the field of PROTACs developed for protein kinases. Since there are already several approved drugs for protein kinases and also numerous selective chemical probes, it is not surprising that this field is also very interesting for the development of PROTACs and has already been used in several studies. The review gives a good overview of the current status of kinase PROTACs and their cellular testing. For some protein kinases, the data of PROTACs based on different E3 ligases are compared.

Besides classical reversible as well as covalent PROTACs, another class has been recently described, the reversible-covalent binding PROTACs. This still new group of PROTACs is the focus of the perspective article by Yuan et al. The authors describe the examples described in literature and discuss the differences in terms of potency, selectivity and duration of action compared to classical PROTACs. Furthermore, the authors give an outlook on the further application of the concept of reversible-covalent binding PROTACs.

In summary, as guest editors, we hope that with the help of all authors and contributors we have compiled useful articles in the field of PROTACs that will also be of interest to a wide range of readers from life sciences. The development of PROTACs is still in its early stages and it is expected that this field will continue to develop dynamically. The results of initial clinical trials with PROTACs in the field of cancer therapy could encourage further expansion.

